# Mechanistic Insights, Treatment Paradigms, and Clinical Progress in Neurological Disorders: Current and Future Prospects

**DOI:** 10.3390/ijms24021340

**Published:** 2023-01-10

**Authors:** Saad Alkahtani, Norah S. AL-Johani, Saud Alarifi

**Affiliations:** Department of Zoology, College of Science, King Saud University, P.O. Box 2455, Riyadh 11451, Saudi Arabia

**Keywords:** neurodegenerative diseases, central nervous system, Alzheimer’s disease, Parkinson’s disease, blood–brain barrier, oxidative stress

## Abstract

Neurodegenerative diseases (NDs) are a major cause of disability and are related to brain development. The neurological signs of brain lesions can vary from mild clinical shortfalls to more delicate and severe neurological/behavioral symptoms and learning disabilities, which are progressive. In this paper, we have tried to summarize a collective view of various NDs and their possible therapeutic outcomes. These diseases often occur as a consequence of the misfolding of proteins post-translation, as well as the dysfunctional trafficking of proteins. In the treatment of neurological disorders, a challenging hurdle to cross regarding drug delivery is the blood–brain barrier (BBB). The BBB plays a unique role in maintaining the homeostasis of the central nervous system (CNS) by exchanging components between the circulations and shielding the brain from neurotoxic pathogens and detrimental compounds. Here, we outline the current knowledge about BBB deterioration in the evolving brain, its origin, and therapeutic interventions. Additionally, we summarize the physiological scenarios of the BBB and its role in various cerebrovascular diseases. Overall, this information provides a detailed account of BBB functioning and the development of relevant treatments for neurological disorders. This paper will definitely help readers working in the field of neurological scientific communities.

## 1. Introduction

Mental health and neurodegenerative illnesses are one of the major groups of noncommunicable diseases (NCDs) that affect people’s quality of life (their thoughts, emotions, behaviors, and relationships) [1,2]. There is no health without mental health. As discussed in a recent world mental health (WMH) report, published by the World Health Organization (WHO) [3], the Kingdom of Saudi Arabia (KSA) became the first country from the Gulf Cooperation Council states to launch a national research-based mental health survey, the Saudi National Mental Health Survey (SNMHS), in collaboration with a national research-based association [4]. In line with earlier reports, mental disability in KSA is similar to that in the rest of the world. Therefore, scientists are engaged in intense research, putting their efforts toward overcoming mental disabilities [5]. This will act as a new milestone to guide national mental health policy, treatment, and diagnosis in the Kingdom of Saudi Arabia [4,6,7].

Neurodegenerative diseases (NDs) are the predominant cause of the gradual loss of neuronal cells, accompanied by cognitive impairment, intellectual deterioration, and often, dementia [8,9,10]. Currently, NDs have garnered significant medical attention because of the gradual rise in cases. These age-related disorders are becoming gradually predominant among people of any age group, mainly because of digital lifestyles, hypertension, hearing impairment, smoking, midlife obesity, depression, physical inactivity, and diabetes [11,12]. Globally, it is projected that nearly 44 million individuals have Alzheimer’s disease (AD) or a related form of dementia [13], and 8.5 million live with Parkinson’s disease (PD) [14]. An estimated 55 million individuals suffered from dementia in 2020 and this figure is predicted to almost double every 20 years, reaching 78 and 139 million by 2030 and 2050, respectively [15]. Treatment of these diseases is difficult; the delivery of drugs to the brain is limited because the blood–brain barrier BBB excludes large-sized drugs [16]. Thus, it is vital to develop a delivery platform that can permit small molecules to access the affected region of the brain without altering the normal physiology of the BBB system [17]. It has been indicated that AD and PD increase consistently and progress with age; the incidence of amyotrophic lateral sclerosis (ALS) increases with age, up to 80 years [18]. Reports from the literature show that AD, PD, and ALS resemble the neurodegenerative actions of malignant brain tumors because of neuronal cell death [19]. In the older age group, above 60 years, diseases such as PD are receiving increased attention from the scientific community [20]. There are many therapeutic drugs that have been employed, either alone or in combination with another drug, to control the symptoms associated with NDs, exhibiting different modes of action (Table 1). These are capable of restoring the balance between the chemicals in the brain, to some extent, in the context of the mild to moderate stages of NDs [21]. For instance, in the majority of PD patients, the chronic long-term use of L-dopa causes motor problems (the on–off phenomenon) and leads to dyskinesia, due to excess dopaminergic tone. Moreover, the metabolism of peripheral L-dopa may result in cardiac arrhythmias, hypotension, and vomiting. Therefore, several approaches have been applied to inhibit the breakdown of L-dopa in peripheral tissues, thereby overcoming the systemic side effects. From this viewpoint, the implementation of the prodrug, carbidopa (a decarboxylase inhibitor), has gained attention in terms of conquering CNS penetration, to minimize the peripheral loss of L-dopa [22]. Accordingly, the levels of L-dopa reaching the BBB will be increased, maintaining near-normal levels of dopamine in the brain [23]. Recently, the effective delivery of drugs into the brain tissue across the BBB has been attained via endogenous receptor-mediated transcytosis (RMT) [24]. Under this circumstance, the nanoparticulate drug carriers or antibodies that target receptors at the BBB, such as transferrin, insulin, and lipoprotein receptors, facilitate their transport across the BBB via receptor-mediated transcytosis (RMT) [25].

NDs such as AD, PD, HD, and ALS arise because of progressive injuries and the loss of neurons [26,27,28]. Different NDs arise when the neurons in the central or peripheral nervous system begin to deteriorate. Indeed, common molecular pathway perturbations, such as oxidative stress, excitotoxicity, mitochondrial dysfunctions, and autophagy impairment have been implicated in the development of the degenerative process in NDs [29]. Researchers have used apoptotic markers to measure neurodegeneration [30,31]. However, neurodegeneration is completely different from apoptosis, based on morphology and time-course progression. Lacking specific molecular markers has been a major hindrance in research into NDs [30]. NDs share the common features and mechanisms associated with the regional aggregation of cytosolic or nuclear proteins [32]. These comprise beta-amyloid (Aβ) plaques in AD, aggregates of α-synuclein in PD and other synucleinopathies, and inclusions of the TAR DNA-binding protein (TDP)-43 in ALS and frontotemporal disorders (FTD) [33].

**Figure 1:**
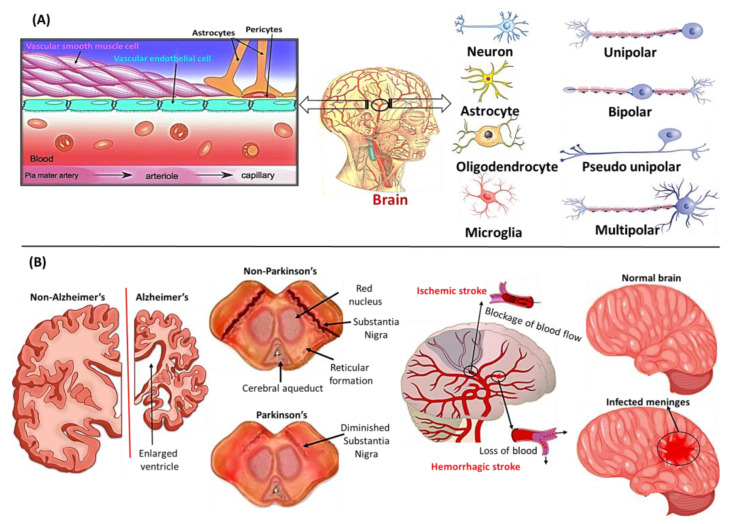
(**A**) A three-dimensional view of the BBB anatomy, composed of vascular endothelial cells, vascular smooth machine cells, pericytes, microglia cells, astrocytes, and various neurons (Source: https://commons.wikimedia.org/wiki/File:Blood_vessels_brain_english.jpg, accessed on 18 December 2022); (**B**) the appearance of different neurodegenerative diseases, such as AD, and PD, ischemic and hemorrhagic stroke, and meningitis-associated damage due to the dysfunction of the BBB. This published work is licensed under a Creative Commons Attribution 3.0.

The central nervous system (CNS) is key for the integration and coordination of signals (electrical and chemical) to regulate the proper movement of the peripheral organs of the body [34]. Normal CNS functioning and development require stringent modulation and a controlled neural microenvironment [34,35,36]. This microenvironment, at the interface between the CNS and circulatory blood, is maintained by the modulation of the efflux and influx of ions, nutrients, waste materials, macromolecules, pathogens, etc. This interface acts as a physical barrier, known as the blood–brain barrier (BBB), which restricts the access of potentially toxic plasma-derived proteins and pathogens into the brain [37,38]. However, peptides and other larger molecules require the expression of specific receptors and/or carriers in the brain endothelium. BBB represents the largest interface for blood–brain exchange, with a projected exchange area of between 12 and 18 m^2^ for the average adult. A nanoscale network of the brain interstitial system (ISS) is made up of continuously connected tubes between the BBB and neurons, allowing the rapid diffusion of molecules across the interstitial space [39,40,41]. Nevertheless, a few blood barriers (retinal, cerebrospinal fluid, and spinal barriers) are also present in the CNS, based on their cellular composition and properties, which plays an important functional role in brain health and NDs. The BBB performs dynamically as a neurovascular unit, comprising neurons, pericytes, microglia, astrocytes, and myocytes (Figure 1) [20]. If dysfunction occurs in any of the neurovascular components, the BBB increases its vascular permeability, enabling toxic molecules and pathogens from the bloodstream to enter the brain and hamper the normal functioning of the brain [36,42]. As a result, neurodegenerative pathways may be activated due to this acute inflammation and weak immune response, which leads to a series of NDs (AD, PD, ischemic and hemorrhagic stroke, and meningitis) (Figure 1) [20,43].

In light of this finding, oxidative damage is revealed as the prime basis of brain-related dysfunction [44,45]. The brain is more susceptible to oxidative stress due to the high degree of oxygen-based free radical generation without appropriate levels of antioxidant protection. The existence of oxidative damage is found to be very high in all NDs. In particular, the protein oxidation of complicated covalent cross-linkages in protein, the disruption of covalent bonds, and changes in different amino acids can lead to mitochondrial damage [35,46]. Among these amino acids, methionine (Met) is more prone to oxidative attack, where it can be transformed into its sulfone products via various mechanisms (H_2_O_2_ treatment) [47]. Several reports have shown that mental disorder arises from the long-term use of pesticides for agricultural demand; this causes widespread toxicity and results in dopaminergic cell death (cell death in the dopamine neurons) [48,49]. Diet-derived natural compounds, including curcumin, berberine, and other phenolic-based candidates, continue to offer possibilities for long-term use in neurological disorders to combat oxidative stress [50,51]. The theme of this review constitutes an overview of the processes involved in the modulatory role of oxidative stress in neurological problems. This review will deliver a common outline of the origin, types, and modes of treatment related to neurological disorders. Furthermore, it will thoroughly discuss the neurovascular unit (NVU), the individual CNS microenvironment, and the functional role of the BBB in the brain, including how its dysfunction leads to various kinds of ND_S_.

**Table 1:** List of the therapeutic drugs used in the treatment of NDs and their modes of action.

NDs	Drug/Bioactive Molecule	Mechanism of Action	Ref.
PD	Donepezil	Acetylcholinesterase inhibition	[52]
	L-dopa and carbidopa	Dopaminergic agonism	[53,54]
AD	Bexarotene	Dissolution of Aβ plaques	[55]
	Memantine	N-methyl D-aspartate receptor antagonism	[56]
	Lacenemab	Reduce Aβ protofibrils in the brain and CSF	[57]
Ischemic stroke	Semagacestat	γ-Secretase inhibitor	[58]
	Aspirin and Statin	COX inhibition and cholesterol synthesis, respectively	[59,60]
Multiple sclerosis	Alteplase	Tissue plasminogen activation	[61]
	Mitoxantrone	Lymphocyte apoptosis	[62]
	Laquinimod	Shifting the T cell response to T helper 2 type	[63]
Glioblastoma	Temozolomide	DNA alkylation followed by apoptosis	[64]
Spinal muscular atrophy	ZD1839 (gefitinib), OSI774 (erlotinib)	Protein kinase inhibition	[65]
	Zolgensma	Replacing the non-working survival motor neuron 1 (SMN1) gene	[66]

## 2. Origin of NDs in Relation to the Blood–Brain Barrier

**Figure 2:**
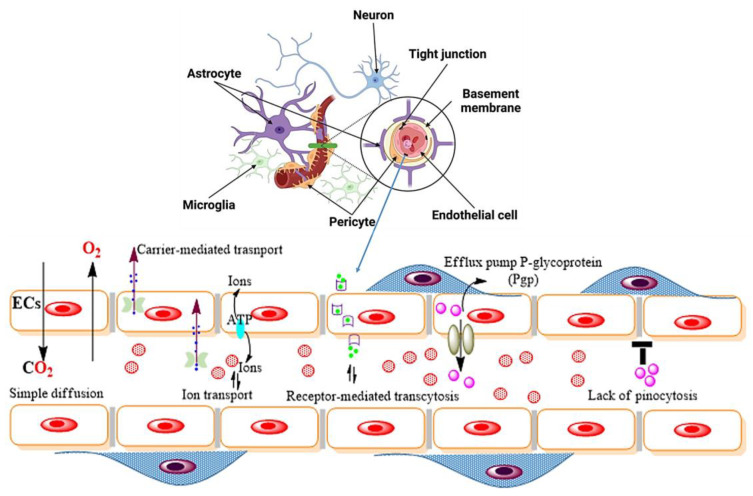
Schematic representation showing the various modes of transport system across the BBB includes carrier-mediated transport (CMT), receptor-mediated transport (RMT), efflux transporters, and ion transport, which are mainly governed by specialized endothelial cells of the BBB. Permission has been taken from the author and this work is licensed under a Creative Commons Attribution 4.0 International License Copyright © 2022. All rights reserved [68].

The specific causes of NDs include genetic and congenital abnormalities, infections, lifestyle, or environmental problems, including malnutrition, and nerve injury. Especially, the NVU plays a key role in maintaining the neurological balance because they sustain the blood–brain barrier (BBB) and regulate the local immune response in the brain. Therefore, dysregulation in the maintenance of the blood vessels within the NVU may lead to a wide spectrum of NDs. Hence, for a healthy brain, there is a need for a balance in the physiological communication between the individual CNS microenvironments [67,68,69]. The BBB is a physical barrier separating the CNS from the systemic circulation, comprising the continuous brain microvascular endothelium, its underlying basement membrane, the pericytes that tightly encircle the endothelium, and the adjacent tissue space made up of astrocytes (Figure 2) [68]. The BBB avails itself of several transport systems, such as carrier-mediated transport (CMT), receptor-mediated transport (RMT), efflux transporters, and ion transporters, by which ions or small molecules can pass into the CNS (Figure 2). These cells regulate the permeability between the blood and the brain compartments in a selective manner. All three cell parts are necessary for the normal physiology of the neurovasculature system and BBB integrity [70,71]. A large number of receptors, as expressed by astrocytes, secrete soluble factors upon activation; these mediate both the innate and adaptive immune responses that act against pathogenic microbes or molecules [72,73]. Brain pericytes have likewise been demonstrated to respond to inflammatory stimuli, causing the discharge of pro-inflammatory cytokines, vigorously playing a part in neuroinflammatory reactions [74]. However, the complex interaction between these cell types and the microvascular endothelium makes it difficult to analyze their individual contribution to neuroinflammation in vivo [69,75]. Moreover, reactive astrocytes not only limit the entry of peripheral substances into the CNS but also trigger a defense mechanism by up-regulating classical tight-junction proteins (TJ proteins) and the junctional adhesion molecules (JAMs) [76].

### 2.1. Endothelial Cells (ECs)

ECs are a key part of the blood vessels and maintain cardiovascular homeostasis by regulating blood fluidity and fibrinolysis, angiogenesis, vascular tone, platelet aggregation, and leukocyte adhesion [77]. The typical vascular endothelium exhibits a cardioprotective effect; however, its irregularity causes a plethora of cardiovascular ailments, including hypertension, atherosclerosis, diabetes, obesity, and aging [78]. In the case of the BBB endothelium, ECs are primary building blocks that form a thin membrane connected to each other via extreme TJs. Due to these TJs, the connection between ECs at the BBB is 50–100 times stronger than ECs at the peripheral wall of the micro-vessel [17]. ECs form part of the BBB, which regulates the entry of blood pathogens, cells, and macromolecules into the CNS. The regulation of CNS nutrients, ions, and energy metabolite transportation is carried out with the help of the brain endothelium. Hence, EC dysfunction eventually leads to the dysregulation of cerebral blood flow and BBB damage; as a result of increased vascular tone, stroke, oxidative stress and further thrombovascular problems in the brain may occur [79]. Owing to their peculiar characteristics, ions or small molecules (e.g., iron or glucose) are passed across the BBB by enzyme-assisted active transporter proteins [17,80,81].

### 2.2. Pericytes

Pericytes are fibroblast-like cells situated in the NVU between ECs, astrocytes, and neurons. Thus, interactions between pericytes and ECs are important for the protection of the BBB [82]. Pericytes receive coordination and process signals from their neighboring cells to mediate diverse neurovascular functions. In the CNS, they are important for the regulation of capillary hemodynamics, BBB permeability, and immune cell entry, all of which are essential for normal brain homeostasis [83]. Interruption of this functionality may lead to the loss of BBB integrity, which is an early sign of neurodegenerative and inflammatory situations in the brain. Pericytes express various contractile and cytoskeletal proteins (e.g., α-smooth muscle actin (α-SMA), vimentin, desmin, myosin, and nestin) and cell-surface antigens (e.g., transmembrane chondroitin sulfate proteoglycan NG2, platelet-derived growth factor receptor-β (PDGFRβ), aminopeptidases A and N (CD13), and the regulator of G-protein signaling-5 (RGS5), CD146), some of which are also found on vascular smooth muscle cells (VSMCs) [84]. Pericytes also communicate with other components of the NVU (e.g., endothelial cells, astrocytes, and neurons) via the autocrine and paracrine signaling pathways to control the neurovascular functions [85,86,87].

### 2.3. Astrocytes

Astrocytes are specialized glial cells that cover the entire CNS and offer essential physiological support for neurons, thereby evolving as modulating factors in diverse NDs [88]. During structural injury within the CNS, astrocyte cells undergo reactive astrogliosis, which helps in restoring CNS homeostasis and limiting tissue damage. Moreover, astrocytes can also elevate the level of TJ proteins by expressing pentraxin 3 and hinder the differentiation of pericytes by interacting with the integrin α2 receptor through the brain-deriving basement membrane of a specific protein (e.g., laminin). Both functions are necessary to maintain low permeability and BBB integrity [84]. Astrocytes regulate the antioxidant glutathione for storing and releasing nutrients to neurons [89]. Besides this, astrocytes act as a regulator for water transport through the aquaporin-4 channel across the cell membrane. It is also assumed that the astrocytes secrete soluble factors that induce the formation of TJs for regulating blood flow in the brain [36,90,91].

### 2.4. Microglia

Basically, microglia cells are the primary innate immune cells of the brain and play an essential role in mediating cerebral homeostasis. They respond to any kind of pathology via microglial activation. Microglia are monocyte lineage cells positioned all over the brain and spinal cord that contain ~5–20% of the total density of the glial cell in the brain parenchyma [92]. Basically, the resident macrophage cells perform two major functions, which are CNS maintenance and immune defense. Furthermore, increasing information within the literature indicates that activated microglia can modulate the expression of TJs to enhance the integrity and functions of the BBB. During the course of chronic neuroinflammation, microglia cells are activated in all brain regions where aggregated α-synuclein (α-syn) accumulates, representing the key pathological hallmark of synucleinopathies [93]. Thus, microglia are exactly adapted to sense many types of danger and differentially react with an alternative reparative response [94].

## 3. Types of Brain Disorders

Neurodegenerative disorders are a collection of debilitating conditions, mainly involving the progressive loss of function and the death of neurons in the CNS [95]. Most neurons are incapable of reproducing to replace lost cells, so this damage is often cumulative and permanent. Furthermore, the most common pathogenetic mechanism involves the aggregation and deposition of specific misfolded proteins, which causes neuronal dysfunction and neuroinflammation. While the type of aggregated proteins with fibril formation and amyloid deposition varies from disease to disease [96], the type of proteins more often than not represents the hallmark of each ND.

### 3.1. Alzheimer’s Disease (AD)

AD is a progressive neurodegenerative disorder that modifies expressed cognition. The onset of symptoms usually begins at 20 years old or above, with insignificant aberrations in the brain that are undetectable in the affected person. It causes severe depression in elderly patients (>65 years old) and eventually leads to death. At this age (> 65 years old), the individual exhibits the symptoms of dementia due to AD, commonly called Alzheimer’s dementia [97]. AD is characterized by personality changes and reduced cognitive function, memory, and language skills, as well as some brain alterations that progressively deteriorate over time [98]. Over time, AD causes a loss of function regarding even the most basic everyday activities [99]. In a number of preclinical and clinical investigations, medicinal plants have been found to have anti-AD efficacy [100,101]. With an increase in age in an AD-diagnosed person, the re-absorption of cerebrospinal fluid (CSF) back into the circulation is reduced. The post-mortem examination of brain tissues from AD patients shows signs of deterioration in the pericytes. Furthermore, plasma platelet-derived growth factor-BB (PDGF-BB) levels are shown to be elevated in AD-diagnosed patients, reflecting pericyte injury, which is also elevated in CSF in mild dementia conditions and is, therefore, potentially useful as a protein biomarker in AD [83]. According to the neurovascular hypothesis, BBB shows the spatial arrangement of amyloid β peptide (Aβ), leading to a rise in the amyloid burden in the growing brain of AD [10]. The Aβ is a key initiator that activates the progression of AD via accumulation and aggregation. AD patients exhibit a shortage of acetylcholine, which is responsible for the transmission inside the brain. There are two major paths of neurotoxicity in AD pathogenesis: free radical generation and Aβ oligomer formation. The excessive reactive oxygen species may be as a result of mitochondria dysfunction and an unusual accumulation of transition metals, whereas the aggregation of Aβ and tau proteins leads to a redox imbalance, thereby developing a malicious cycle that induces neurotoxicity and the progression of AD [102]. Animal studies have shown that the level of Aβ decreases with the administration of antisense oligonucleotide, directed at amyloid precursor protein (APP), and it is capable of crossing the BBB [103]. As a result, it restores Aβ spatial arrangement and cognition in AD, because of the fact that APP plays a significant role in the sequential proteolytic cleavages that result in the generation of Aβ-peptides. Mutations in APP genes such as presenilin-1 and presenilin-2 and APO-ε4 are implicated in the pathology of AD [104].

**Figure 3:**
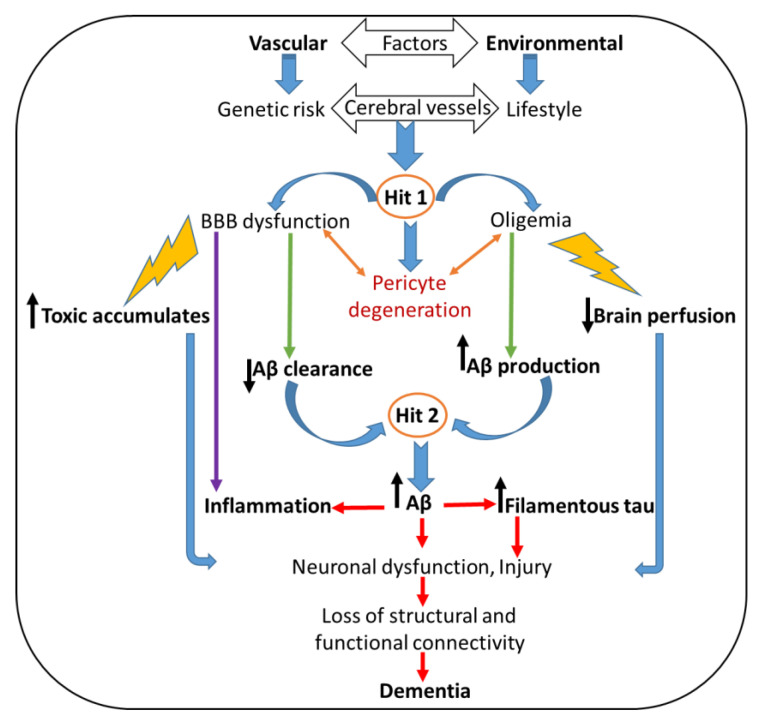
Replotting of a neurodegeneration pathway involved in AD pathogenesis, mediated by elevated levels of Aβ production, can independently and/or synergistically lead to the formation of filamentous Tau pathology [105].

AD pathogenesis is influenced by various risk factors, including genetics, vascular conditions, environment, and lifestyle. A schematic representation of the two-hit neurovascular hypothesis for ADs has been summarized in Figure 3 [105]. According to this theory regarding AD, cerebrovascular dysfunction and disruption in the neurovascular integrity occur via two routes: (i) Aβ-independent (hit 1), which directly causes neuronal injury, and (ii) Aβ-dependent (hit 2), which accelerates neurodegeneration and cognitive impairment. In the latter case, BBB dysfunction leads to the faulty clearance of Aβ from the brain, whereas reduced brain perfusion increases Aβ production, causing Aβ accumulation and neuronal injury. In combination, reduced brain perfusion (Hit 1) and elevated levels of Aβ (Hit 2) have a striking synergistic effect on neuronal Tau phosphorylation and pathology, leading to faster damage to the neurons.

The pathogenesis of AD is indicated by the occurrence of neurofibrillary tangles and neuritic plaques. Insulin has long been a matter of concern in AD because it mediates the dysregulation of bioenergetics and processes to AD as a mechanistic link between them [106]. The insulin receptor is frequently expressed in the CNS; it is involved in neuronal development and cognitive activity. Remarkably, defects in insulin signaling in the brain lead to AD [105]. Insulin inside the brain has been shown to control both peripheral and central glucose metabolism and be neuroprotective. Recently, CNS insulin resistance has been shown to play a role in the progress of dementia and AD due to the reduced CSF-to-plasma insulin ratio which hampers the transport of insulin across the BBB [107].

### 3.2. Treatment of Ads

Treatments of Ads involve two major therapeutics: (i) cholinesterase inhibitors, and (ii) β-amyloid targeted drugs.

(i) Cholinesterase inhibitors (ChEIs)

Acetylcholinesterase (AchE) inhibitors, which increase the availability of acetylcholine at cholinergic synapses, are one of the most valuable approaches for the treatment of Ads [108]. The cholinergic system contains the neurotransmitter molecule, acetylcholine (Ach), which acts as a neurotransmitter in the central nervous system (CNS) and peripheral nervous system (PNS). It is crucial for neuronal function in memory, learning, and other vital features of cognition; therefore, its deficiency leads to dementia in patients with AD. Cholinesterase inhibitors (ChEIs) are administered to palliate cholinergic deficiency by blocking the action of cholinesterase, which is responsible for the breakdown of acetylcholine. Commonly used FDA-certified drugs are rivastigmine, memantine, donepezil, and galantamine, which act as ChEIs to treat the different phases of AD [109]. ChEIs work by decreasing abnormal activity in the brain. Memantine may improve the ability to think and remember by increasing the number of neurotransmitters in the brain. Moreover, memantine blocks certain receptors from excess stimulation, which can injure nerve cells [97]. These drugs support communication between nerve cells. Nevertheless, the strength of ChEIs is significantly reduced by the presence of the BBB. Therefore, the implementation of biocompatible nano-carrier systems, such as chitosan-coated PLCGA NPs, has been conjugated with novel anti-Aβ antibodies that are employed to transport these drug candidates beyond the BBB [110,111]. The advantage of this strategy is that the nanoparticles are small in size (1–100 nm) and can easily cross the BBB and/or produce damage to the barrier’s integrity by altering endothelial cell membrane permeability.

(ii) Amyloid-β-targeted drugs

Aβ is the major pathologic protein in AD and its deposition is an important factor in AD prognosis. This is because the neurotoxic effects induced by Aβ, due to the presence of various amino acids, weaken the blood–brain barrier (BBB). Hence, AD development is linked to BBB dysfunction and Aβ deposition. The specificity of the CNS is greatly enhanced by bio-therapeutic proteins, peptides, and monoclonal antibodies under a nanotechnologically based drug delivery system that provides a unique function for restoring damaged cells. At present, there is an FDA-approved monoclonal antibody (aducanumab) being directed against Aβ for the treatment of AD [112]. Mechanistic investigation showed that the brain microvascular endothelial cells (BMEC) of the BBB regulate the antibody for the delivery of bioactive molecules to the CNS, with the help of transferrin [113]. Salvati et al. examined the affinity of liposomes (LIP) functionalized with phosphatidic acid (PA-LIP) toward Aβ and were able to rescue cells from Aβ toxicity in vitro. The mechanistic investigation showed that the PA-LIP conjugated system binds with the anti-transferrin RI7217 monoclonal antibody (RI7217 mAb) versus biotin/streptavidin ligation, to facilitate liposomes in overcoming the BBB [114]. Similarly, Kogan et al. removed the deposition of Aβ protein aggregates and plaques involved in AD by molecular surgery, using the local heat dissipated by gold nanoparticles (AuNP). In this case, AuNPs, linked to the peptide AuNP-Cys-PEP conjugates attached to the fibrils, absorbed the radiation and dissipated the energy, causing disaggregation of the amyloid aggregates under a weak microwave field (0.1 W) [115].

### 3.3. Parkinson’s Disease (PD)

**Figure 4:**
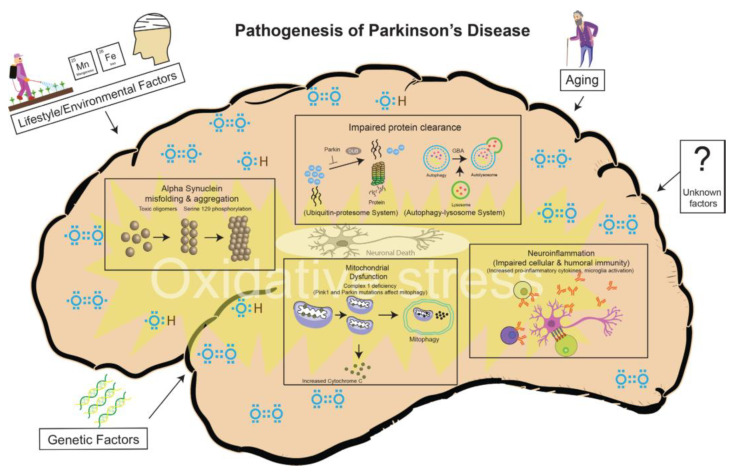
Pathogenesis of PD—a variety of cellular mechanisms, with a background of oxidative stress, coupled with aging, lifestyle/environmental, and genetic factors, contribute to PD-related neurodegeneration. Permission was taken from the original authors for using this diagram [120] and licensed under a BMJ Publishing Group Ltd. and Copyright Clearance Center 2020.

After AD, PD is the second most common neurodegenerative disorder, and it is also a progressive disease that worsens with time. Both genetic and non-genetic stimuli cause PD, although age is considered the primary risk factor. Moreover, several other factors, such as too high a caffeine intake, smoking, and environmental toxins are recognized for the development of PD (Figure 4), although the exact mechanism remains unclear. The symptoms of PD that are termed parkinsonism (rigidity, resting tremor, postural instability, and bradykinesia) are differentiated clinically and pathologically by the loss of neurons and the deposition of ubiquitinated protein in the neuron’s cytoplasm (also known as Lewy bodies) [116]. Cross-sectional neuropsychology studies have revealed that the older age of disease onset is related to more severe cognitive impairment in PD-diagnosed persons. According to epidemiologic findings, the age-specific incidence of dementia in PD ranges from 12.4% in those aged 50–59 years to 68.7% in those aged more than 80 years [117,118]. About 15 percent of people with PD have a family history of the condition, and family-linked cases can result from genetic mutations in a group of genes, including the *LRRK2, PARK2, PARK7, PINK1*, or *SNCA* genes. The neuronal damage initiated by inflammatory responses is linked to cognitive and motor deterioration, which contributes to the breakdown of the BBB [54]. PD treatments are aimed at restoring the levels of dopamine or correcting the functional perturbations of the basal ganglia, caused by the loss of dopamine. Both motor dysfunction and cognitive decline are peculiar properties of Lewy body dementia (LBD) and are caused by the accumulation of the α-synuclein protein in neurons. Synaptic dysfunction and a low level of dopamine are frequently seen in PD patients [119,120].

### 3.4. Treatment of PD

The enzyme monoamine oxidases are responsible for eliminating the neurotransmitter, dopamine; therefore, their inhibition enhances the bioavailability of dopamine. Some of the drugs that are widely used in the treatment of PD include dopamine agonists, L-dopa, and monoamine oxidase B inhibitors. L-dopa is a prodrug that is converted to dopamine by DOPA decarboxylase for the stimulation of dopaminergic receptors [121]. Hence, L-dopa is the immediate precursor to dopamine and crosses the blood–brain barrier to increase dopamine neurotransmission, thereby compensating for the depleted supply of endogenous dopamine in Parkinson’s disease. Another dopamine-receptor agonist, apomorphine, has been used in the early-stages treatment of PD. Recently, gene therapy methods for the treatment of PD include: (i) neurotrophic factors, and (ii) proteins that work to overdraw dopamine production [122].

(i) Neurotrophic factors (NTFs)

NTFs are a group of neuropeptides (such as nerve growth factors) that support the development, maturation, and survival of neurons in the CNS. Nevertheless, NTFs prevent the loss of dopaminergic neurons and play a potential role in the treatment of PD [123]. However, the introduction of NTFs directly into the brain is limited, whether in terms of the cost issue, risky intracranial surgery, poor diffusion, and/or the failure to pass through the BBB. To circumvent these issues, the progress of nanoparticles that cross the BBB is of prime importance for the treatment of PD. For effective NTF delivery through the BBB, the surface of NPs can be functionalized with specific ligands, such as transferrin, insulin, lactoferrin, apolipoproteins, antibodies, or short peptides that will be recognized and internalized by the respective receptors on the brain endothelial cells [123]. These factors can either enhance or upregulate the expression of specific genes or even restore the normal function of the gene products. In this procedure, a nanocontainer, such as a nanoparticle or a liposome, encapsulates the plasmid DNA (pDNA) in order to deliver its payload into the cells of the central nervous system. NTFs facilitate the genetic repair of injured dopaminergic neurons to slow down neurodegeneration in PD, using the BNDF gene (brain-derived neurotrophic factor—a protein-coding gene) via the neurotrophic factor-like glial cell line-derived neurotrophic factor (GDNF). It is worth mentioning that once the vectors enter the targeted cells, the transport of pDNA into the nucleus still needs to overcome the intracellular barriers. These barriers contain a lipid bilayer to obstruct hydrophilic pDNA trafficking. Here, the lysosome degrades the pDNA, which delays pDNA diffusion in the cytoplasm. Therefore, a gene delivery approach that can simultaneously overcome the BBB and intracellular barriers in the brain area is greatly needed [124].

An emerging method of intracellular drug delivery is a cell-penetrating peptide (CPP), termed “the Trojan horse approach”. This is based on the receptor-specific monoclonal antibody (mAb) that is conjugated onto the nanomedicine surface. The Trojan horse occupies specific receptors exposed on the BBB, and the brain cell membrane, to initiate the transport of the nanomedicine from the blood into brain cells beyond the BBB [125,126,127].

(ii) Proteins that work to overdraw the dopamine production

Dopamine synthesis begins in the nerve terminal with the amino acid, phenylalanine, and proceeds sequentially through its conversion to tyrosine, which is transported across the BBB. Hence, the insertion of these amino acids leads to an increase in the levels of dopamine produced in the nerve terminal. Xia et al. examined the intravenous vector administration of Trojan horse liposomes (pegylated immunoliposomes) and monoclonal antibodies in the animal model of PD. The study showed that the successful transport of therapeutic plasmid-containing DNA for GDNF across the BBB is facilitated by the transferrin receptor [128]. They also incorporated the gene promoter for tyrosine hydroxylase (TH), a key enzyme for the conversion of tyrosine to dopamine, to limit the expression of the transgene to catecholaminergic neurons. Another technique that has also been of interest is the nanoparticle-mediated endogenous delivery of catalase to decrease microglia-activated ROS generation in the PD brain. More recently, metal-oxide NPs (CeO_2_-NPs) exhibiting antioxidant activity have been applied clinically for the restoration of dopamine [129,130].

### 3.5. Huntington’s Disease (HD)

HD is an inherited brain disorder caused by a mistake in the DNA instructions. This defect is “dominant”, meaning that anyone who inherits the mutation from parents with Huntington’s disease will ultimately develop the disease. In addition, brain changes in Huntington’s disease lead to changes in mood, especially depression, anxiety, and uncharacteristic anger and irritability. Obsessive-compulsive behavior is also common, causing a person to repeat the same question or activity over and over again [120].

The HTT gene encodes the huntingtin protein, which seems to play an important role in nerve cells (neurons), such as chemical signaling, materials transport, and the protection of nerve cells from self-destruction (apoptosis), which is required for the normal development of the embryonic brain. The mutation in the HTT gene that results in HD is a CAG trinucleotide repeat, which typically is repeated 10 to 35 times within the gene for the huntingtin protein. However, HD patients exhibited a higher number of CAG-repeating units (40–60 times), referred to as mutant huntingtin (mHTT) aggregate. This mutation causes the huntingtin protein to form intracellular inclusions in the brain and peripheral tissues [131]. Astrocytes are more capable of clearing the aggregates than neurons; they are immune to mHTT accumulation. Furthermore, the case of neuronal excitotoxicity (the injury and death of neurons) develops once mHTT aggregates into astrocytes, altering the glutamate signaling in HD [132]. There are several other features of HD appearing without an alteration in glutamate release, one of which is the low expression of the Kir-4.1 gene in astrocytes. Kir-4.1 controls the GLT1-mediated homeostasis and Ca^2+^ signaling pathway [133]. Under these circumstances, astrocytes exhibit a low risk of neurotoxicity and inflammation.

### 3.6. Amyotrophic Lateral Sclerosis (ALS)

**Figure 5:**
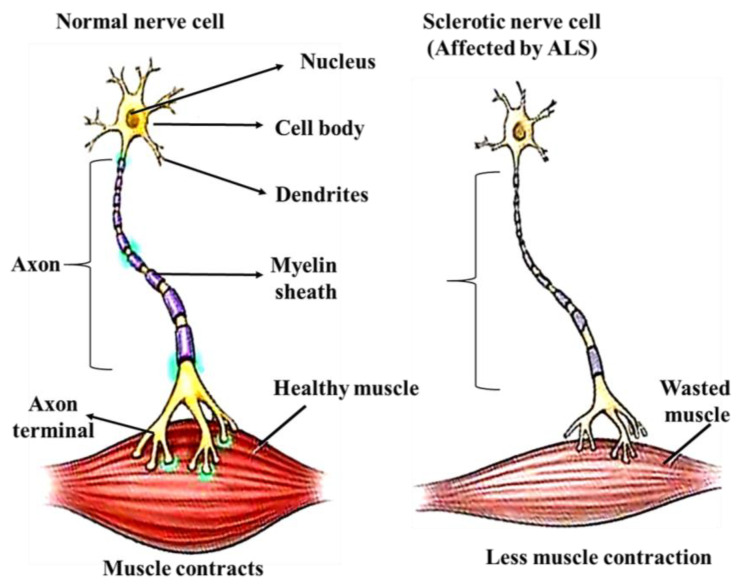
A schematic illustration of muscle dysfunction and ALS pathology. The left panel depicts muscle tissue supplied by a normal nerve cell and the right panel shows the diseased state.

ALS is a rare neurodegenerative disease caused by the loss of those motor neurons responsible for controlling voluntary muscle activity. Early symptoms of ALS are expressed as the progressive weakening of muscles because of the gradual deterioration of motor neurons [134]. Motor neurons are responsible for integrating signals from the brain to the muscles and organs, so their failure leads to a loss of muscle control. A secondary symptom associated with ALS is the development of depression. People with ALS have problems with memory and decision-making and, finally, are diagnosed with a type of frontotemporal dementia [135]. In this type of dementia, behavioral changes are common, with high impulsiveness and repetitiveness, as well as a marked difference in dietary habits [136,137]. To date, many hypotheses have been suggested; however, the mechanisms of neuronal death in ALS include defective glutamate metabolism, free radical injury, mitochondrial dysfunction, gene defects, programmed cell death (apoptosis), etc. [135]. The precise molecular and cellular basis for neuronal death is not yet well established, but the existing view is that it is a culmination of multiple abnormal biological processes [134]. About 90% of ALS cases are classified as sporadic ALS (sALS), which has no obvious genetically inherited component. The remaining 10% of ALS cases are of the familial type (*C9orf72, CHCHD10, SQSTM1,* and the *TBK1* gene). A schematic representation of muscle dysfunction and ALS pathology is depicted in Figure 5 [138].

From a biochemical viewpoint, the heterogeneity of ALS is complex and appears to be a multi-step process at the cellular level, which is characterized by upper and lower motor neuron degeneration stemming from the brain and spinal cord, respectively. Although both upper and motor neuron lesions result in muscle weakness, they are clinically distinct due to various other manifestations. However, the majority of problems such as muscle atrophy, muscle twitching, decreased reflexes and muscle tone, and negative Babinsky signs are associated with lower motor neuron lesions. The major pathological signs of ALS are protein misfolding constituents that are now categorized as “prion-like” due to the aggregation of the misfolded proteins. Hence, it is characterized by protein inclusions formed by either TAR DNA-binding protein (TDP-43), SOD1, or fused in sarcoma (FUS), in both upper and lower motor neurons. Given the contribution of reactive astrocytes, microglia, and compromised oligodendroglial function, ALS should be considered a non-cellular, autonomous disease [135].

**Figure 6:**
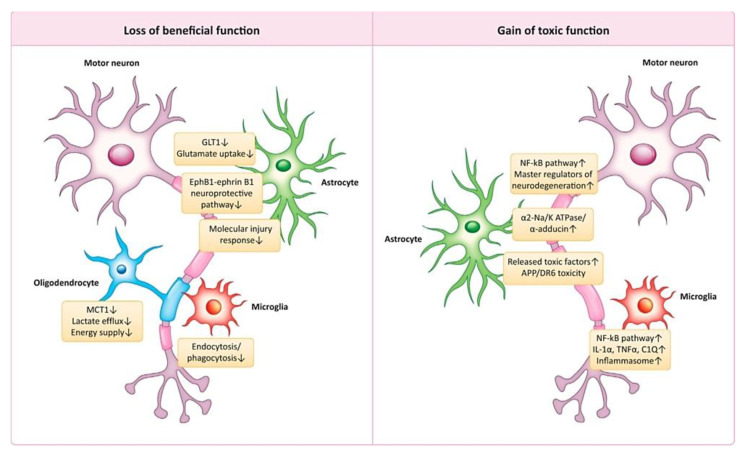
Illustration of the non-cell autonomously based pathogenesis of ALS. This published work is licensed under a Creative Commons Attribution 4.0 International License Copyright © 2021 and permission taken from the original authors for its re-use. All rights reserved [134].

Two mechanistic postulates: a loss of beneficial functions or the acquisition of noxious properties can be proposed to explain the contribution of non-neuronal cells to the demise of motor neurons in ALS (Figure 6) [134].

Unfortunately, ALS has long been considered an incurable disease, with an expected life expectancy of 3–5 years after the onset of symptoms. There are many antioxidants and supplements that have been anticipated as alternative treatments for ALS. These agents protect against the oxidative damage mediated by toxic free radicals, which has been implicated in the pathogenesis of ALS [66], and several antioxidant agents have been tested in patients with ALS, including Vitamin E, N-acetylcysteine, methylcobalamin, glutathione, L-deprenyl, creatine, and coenzyme Q10. Despite decades of passionate investigation, only a few FDA-approved drugs—riluzole, the combination of dextromethorphan HBr and quinidine sulfate, and edaravone—are available for treating ALS.

## 4. Future Perspectives

In the area of BBB study, researchers have expanded their work to identify that the BBB is not merely a barrier that blocks drugs but it also facilitates communication between the brain and peripheral tissues of the CNS. This complexity offers a wide range of opportunities for the development of drug targets to cross the BBB. In the future, many investigations into endothelial–astrocytic interactions and cellular proteomics can pave the way for the design of targeted therapies essential for BBB functions. Finally, variations in age, gender, genetic makeup, and lifestyle may affect the BBB in a delicate manner, so that successful treatments may depend on particular micro-profiling and the improvement of personalized medicine.

## 5. Conclusions

The focus of this review was to gain awareness regarding the existing NDs and the current therapeutic strategies. The BBB is a cellular barrier that regulates the microenvironment around the CNS for the proper functioning of neurons. This barrier becomes a determining factor for the treatments of NDs due to its regulation of particle tracking across this physical barrier. In essence, this function of the BBB is an essential consideration in the improvement of CNS-acting therapeutics. Evidence from the literature supports the finding that neuroinflammation for the CNS arises due to numerous molecular mechanisms, resulting in pathological conditions such as dementia, hypertension, normal aging, stroke, and depression. Furthermore, the dysfunction of the BBB also leads to a neurodegeneration effect. Therefore, to maintain the normal functioning of the brain, it is necessary to maintain BBB homeostasis. Moreover, the significant role of the BBB increases attention in finding new drug delivery platforms to restore normal barrier function.

## Data Availability

Not applicable.
